# Effect of different types of acid-etching agents and adhesives on enamel discoloration during orthodontic treatment

**DOI:** 10.34172/joddd.2021.002

**Published:** 2021-02-13

**Authors:** Arman Mohmmadi Shayan, Ahmad Behroozian, Amirhouman Sadrhaghighi, Amirmohammad Dolatabadi, Sevda Hashemzadeh

**Affiliations:** ^1^Department of Orthodontics, Faculty of Dentistry, Tabriz University of Medical Sciences, Tabriz, Iran; ^2^Dental Student, Faculty of Dentistry, Tabriz University of Medical Sciences, Tabriz, Iran; ^3^Dentist, Private Practitioner, Tabriz, Iran

**Keywords:** Acid etching, Adhesive, Orthodontics, Staining

## Abstract

** Background.** Acid etching and bonding is a routine process in orthodontic treatment. The present study aimed to evaluate enamel discoloration after using different types of acid etching and adhesive agents.

**Methods.** A total of 105 extracted human premolars were divided into six groups regarding the type of acid etching agent: solution and gel of 37% phosphoric acid, and type of self-cured adhesive agent: Unite (3M, fluoride-free), Resilience (Ortho Technology), and Rely-a-Bond (Reliance, fluoride-releasing adhesive), with each group containing 15 specimens. All the selected teeth were subjected to a staining process, and color parameters were determined using a spectrophotometer.

**Results.** The type of phosphoric acid (solution or gel) had no significant effect on the color change of enamel (*P* >0.05). Resilience significantly changed the enamel color compared to the Unite and Rely-a-Bond (*P* <0.001). There was no significant difference in color change between the Unite and Rely-a-Bond adhesives (*P* =0.67). The difference in color change between all the three time intervals (T0-T1, T1-T2, and T0-T2) was significant (*P* <0.001).

**Conclusion.** In this study, the type of phosphoric acid (solution or gel) did not result in any significant difference in enamel color. Also, considering the lack of the effect of the orthodontic adhesive type in terms of fluoride release or no fluoride release, it can be concluded that this is most affected by the commercial brand of adhesives.

## Introduction

Orthodontic treatment can lead to undesirable side effects on the enamel surface,^1,2^ including loss of enamel by the etching process, surface changes, decalcification, and microcracks and scratches during the deboning and polishing processes.^1-5^ These changes, in addition to structural defects, can lead to discoloration and enamel esthetic problems.^3^ Therefore, considering the above conditions, it is necessary to improve bonding properties to reduce enamel defects and discoloration.^5,6^

It has been shown that the enamel bonding process can be an influential factor in tooth color changes.^7^ A significant change in the enamel color has been observed following demineralization.^8^ Acid-etching agents are used for different purposes in dentistry.^9^ It has been reported that the enamel surfaces etched with phosphoric acid solution differed from its gel form so that the acid solution was able to etch better and more uniformly than the acid gel. The acid solution also increased the number and distribution of resin tags on the enamel surface compared to the acid gel. However, these two types of acids did not lead to significant differences in tensile bond strength between enamel and resin.^10^ The effect of this difference on the surface pattern caused by the type of acid etching agent on the enamel discoloration during orthodontic treatment has not been determined. The etched enamel is remineralized, but the time required for this process is different, and the enamel’s return to the original state is not complete.^11,12^

On the other hand, numerous studies have reported the effect of fluoride-releasing orthodontic adhesives in preventing the demineralization of enamel adjacent to orthodontic brackets.^13,14^ Fluoride can form crystalline fluorapatite in the enamel structure, which is less soluble in the oral environment than hydroxyapatite.^15,16^ Kim et al^8^ showed that fluoride application could partially reverse the color change caused by white spot lesions. Therefore, it is hypothesized that fluoride-releasing orthodontic adhesives might cause less discoloration than fluoride-free adhesives, which has not been investigated in previous studies.

## Methods

A total of 105 extracted human premolars were selected and randomly divided into six groups:

- Group 1: 37%, acid-etching solution; adhesive: Unite (3M, USA, fluoride-free)
- Group 2: 37% acid-etching solution; adhesive: Resilience (Ortho Technology, USA, fluoride-releasing)
- Group 3: 37% acid-etching solution; adhesive: Rely-a-Bond (Reliance, USA, fluoride-releasing)
- Group 4: 37% acid-etching gel; adhesive: Unite (3M, USA, fluoride-free)
- Group 5: 37% acid-etching gel; adhesive: Resilience (Ortho Technology, USA, fluoride-releasing)
- Group 6: 37% acid-etching gel; adhesive: Rely-a-Bond (Reliance, USA, fluoride-releasing)

Each group consisted of 15 teeth, and another 15 teeth were considered as the control group.

Inclusion criteria were the first or second maxillary or mandibular premolar teeth diagnosed by observational evaluation. Exclusion criteria consisted of teeth with carious lesions, restorations, color changes, developmental enamel defects, fractures and cracks, white spots, and fluorosis.

### Bonding process

The samples were first washed and cleaned with water and kept in 0.1% thymol solution at 4°C until used in the study. The tooth roots were embedded in self-curing acrylic resin blocks (PMMA) (Acropars Self-cure, Marlik, Medical Co, Iran) 1 mm apical to the cementoenamel junction. The teeth were polished at low speed using fluoride-free pumice rubber caps in a handpiece and then washed with water and completely dried. In this experiment, metal brackets (standard edgewise, American Orthodontics, USA) with 0.02-inch slots were used for premolars. After etching and priming, the brackets were placed 3 mm gingival to the buccal cusp tip at the mesiodistal center of the dental crown and parallel to the long axis of the tooth. After bonding, all the samples were subjected to a staining process. The samples were immersed in a coffee solution (Nescafe Classic, Nestle, Switzerland) at 37°C for one week. The solution, which was changed daily, was prepared by dissolving 3.6 g of coffee powder in 300 mL of distilled boiling water and filtered for 10 minutes using filter paper.^17^ Then, the brackets were removed by bracket-removing pliers (American Orthodontics, USA), and the remaining enamel adhesive was removed by a tungsten carbide finishing bur (12 blades, Dentaurum) in a low-speed contra-angle handpiece (20 000 rpm) without water. Then, the enamel surface was polished using a rubber cup with pumice and water for 30 seconds. Finally, the enamel color was evaluated in two points: 4 mm distal to the buccal cusp tip of the tooth (bracket bonding site, S1) and a point 3 mm gingival to S1 (S2). The color determination was carried out in three stages: before bracket bonding (T0), after bracket debonding and finishing (T1), and after the second stage of the staining process (T2). For this purpose, a spectrophotometer (Vita Echinade, Vita Zahnfabrik, Bad Sackingen, Germany) was used by a skilled operator blinded to the type of adhesive and composite resin used in the samples, under the manufacturer’s instructions. The CIEL*a*b* system was used to determine the enamel color.

The data were analyzed using repeated-measures ANOVA, Tukey LSD tests, independent *t* test, and one-way ANOVA. P<0.05 was considered significant.

## Results

One hundred five sound extracted human teeth were selected. Six groups (n = 15) of teeth underwent acid etching, bonding, and staining processes. Two examiners carried out the color evaluation with a strong intra-class correlation (Table 1). Color parameters, i.e., L, a, and b, showed a strong correlation, too (Table 2). No significant difference in color change was noted between the acid-etch solution and gel (*P* > 0.05). The color changes were significant between T0-T1, T1-T2, and T0-T2 (*P* < 0.001). Regarding the adhesive type, Resilience (Ortho Technology, USA, fluoride-releasing) resulted in more color change compared to other groups (*P* < 0.05).

**Table 1:** Correlation between two examiners

**Average measures**	**Lower bound**	**Upper bound**	**Inter-rater consistency**	***P*** **value** ^a^
Lightness	0.94	0.98	0.97	0.001
Red-green parameter	0.95	0.99	0.98	0.001
Yellow-blue parameter	0.98	0.99	0.99	0.001

^a^ *P*<0.05 was considered significant

**Table 2:** Correlation between tcolor factors

**Average measures**	**Lower bound**	**Upper bound**	**Inter-rater consistency**	***P*** **value** ^a^
Lightness	0.95	0.98	0.92	0.001
Red-green parameter	0.99	0.99	0.98	0.001
Yellow-blue parameter	0.96	0.98	0.92	0.001

^a^ *P*<0.05 was considered significant

## Discussion

Orthodontic treatment can cause various side effects on the enamel,^1,2^ including enamel loss during etching and decalcification and microcrack formation during debonding and polishing processes.^1-5^ It has been demonstrated that bonding to the enamel can cause tooth discoloration.^7^ Tooth discoloration is a major concern in fixed orthodontic treatment. Furthermore, demineralization, which is seen in orthodontic treatment, can change the enamel color.^8^ This in vitro study compared the effect of different etchant types and fluoridated or non-fluoridated adhesives on the enamel discoloration. The null hypothesis of this study that the type of etchant and fluoridation of the adhesive does not affect enamel staining was confirmed. This study showed that the effect of staining becomes stronger over time.

Discoloration of composite resins is caused by extrinsic and intrinsic factors. One of the extrinsic factors is staining by adsorption or absorption of coloring agents.^18^ The presence of residual adhesive resin tags in the enamel surface can cause direct absorption of exogenous stains, resulting in tooth discoloration.^19^ This phenomenon suggests that the type, depth of penetration, and chemical composition of these resin tags can play an important role in enamel discoloration. Legler et al^1^ showed that etching duration and concentration could influence the curing depth.However, we could not find any significant difference between acid-etching gel and solution.

Raji et al^20^ evaluated the effect of fluoride release from orthodontic adhesives on nanomechanical properties of the enamel around orthodontic brackets and showed that the fluoride-releasing ability of adhesive agents could have some effects on the hardness and elastic modulus of enamel.^20^ They proposed that the fluoride released from adhesives could return basic characteristics to enamel after the etching process. Basdra et al^13^ reported that fluoride released from orthodontic adhesives alters the enamel surface and might inhibit enamel demineralization. Although numerous studies demonstrated the positive effect of fluoride-releasing orthodontic adhesives on preventing demineralization of the enamel around the brackets,^14,20-22^ there is no clear evidence of the effect of fluoridated composite resins in preventing enamel discoloration around metallic orthodontic brackets. The present study showed that fluoridated agents did not significantly affect the discoloration susceptibility of enamel. Karamouzos et al showed that fixed orthodontic treatment on its own could cause color changes in teeth.^23^ Some studies demonstrated that different bonding agents had different amounts of resin penetration; therefore, it is plausible that they can cause different effects on staining susceptibility of the enamel.^6^ The present study showed that the effect of staining was more prominent in Resilience (Ortho Technology, USA, fluoride-releasing) compared to other groups.

We suggest additional studies with microscopic evaluation of the actual amount of resin penetration. Also, we propose randomized clinical trials to mimic the real staining condition of the oral cavity instead of in vitro staining.

## Conclusion

Type of acid-etching agent and fluoride-releasing capability of the adhesive cannot influence the degree of discoloration of the enamel during fixed orthodontic treatment.

## Authors’ Contributions

AMS initiated, conceptualized, and supervised the research work. AMS, AS, and SH prepared the samples and performed experiments with the collaboration of AB. All authors have contributed to analyzing the data and writing the manuscript.

## Acknowledgments

We would like to acknowledge the Dental Materials Center of the Faculty of Dentistry for their assistance during the laboratory tests.

## Funding

This work was supported by Vice Chancellor for Research, Faculty of Dentistry, Tabriz University of Medical Sciences, Tabriz, Iran.

## Competing Interests

The authors declare no competing interests.

## Ethics Approval

This research was approved by the Research Ethics Committee of the Faculty of Dentistry (IR.TBZMED.REC.1395.1003).
